# Sea Sickness—Therapeutic Indications

**Published:** 1896-05

**Authors:** H. N. Guernsey


### SEA SICKNESS—THERAPEUTIC INDICATIONS.

H. N. GUERNSEY, M. D.

*Arsenic.*—Same thirst as of Phosph., but the substances are vomited *immediately.* Feels *very* weak and *exhausted* after vomiting.

*Bellad.*—Nausea, with retching and gagging, but not much vomiting.

*Bryonia.*—Wishes to keep still; the least *motion*, sitting up, moving about, even the motion of the hand, may aggravate the nausea or vomiting. All food is ejected at once.

*Cocculus.*—When one is satisfied that the motion of the vessel aggravates the nausea, and produces vomiting. Headache, with a strange feeling in the head, a sort of uncertainty.

*Ipecac.*—Constant sensation of nausea, vomiting without any relief.

*Nux-vom.*—Nausea, with a sensation as if vomiting would bring relief; it seems as if some substance were in the stomach creating the nausea, which, if ejected, would cure.

*Phosp.*—Thirst for cold water and for cold substances generally, which after awhile (as soon as warm in the stomach), increase the nausea, and are vomited.

*Pulsat.*—Nausea and vomiting, with a very bad taste in mouth, and of all substances ejected; one wishes to cleanse the mouth with cold water frequently; a slimy, sticky feeling in the mouth.

*Secale.*—Nausea, with a sensation as if too warm, one wishes less clothing; finally vomiting profusely, with a marked sense of relief; one feels *much* better every way for awhile, then the same scene is repeated, and so on.

*Sepia.*—Painful sensation of emptiness at pit of stomach; smell of food aggravates the nausea.

*Tabac.*—Nausea, with great sense of weakness and faintness; can hardly stand or sit up, a deathly sort of feeling; cold sweat.

